# Loneliness, its effect on mental and physical health, and the dying

**DOI:** 10.1177/02692163221135223

**Published:** 2022-11-15

**Authors:** Ami Rokach

**Affiliations:** York University, Toronto, ON, Canada

Loneliness, a universal experience shared by every human. It is, in fact, the first negative thing that the biblical God named was loneliness which is related to numerous somatic, psychosomatic, and psychiatric phenomena and disturbances.

Ernst et al.^1^ reviewed the effects of COVID-19 on loneliness and asserted that “social isolation and loneliness were becoming major public health and policy concerns, largely due to their serious impact on longevity, mental and physical health, and well being” (p. 661). There are several theoretical views regarding what loneliness is, its causes, and its meaning. Based on my 40 years of research, the following characteristics of the experience of loneliness are delineated (see also Rokach^2^):

1. *Loneliness is an experience of separation*, which is familiar to every human, who has undergone it at one or more stages of their lives.
2. *Loneliness may arise at birth or in childhood and be present throughout the person’s life.*
3. *It is difficult to tolerate*. Loneliness, unlike solitude, is painful, unwanted, and inevitably causes suffering.
4. *Evolutionarily speaking, loneliness motivates humans to seek meaning and connection*, for without a community we cannot survive.
5. *Loneliness may bring about personal growth and new possibilities*. Under some conditions, resilient people who experience loneliness may be motivated to grow, explore, come out of their shells, and connect with those who they now appreciate as the supporters they need.

## Medical approach to death and dying

Modern medical science has been focused, even obsessed, with death, which provided medicine with a clear “enemy,” and consequently, medical research has focused on conquering the diseases that cause death. Only in the last half century are physicians becoming more aware of the necessity of approaching and treating the patient as a person, rather than a disease. For those who are dying, and [almost always] these are the times they are most dependent on the support of those they love and on their circle of friends and family. Abel and Kellehear^3^ highlighted the importance of satisfying social relationships and their positive influence on health and wellbeing, a fact that the public health system must be aware of and condone. Palliative care aims to address that sorry issue, offering the best quality of life for the dying, their families and support people. It is geared to providing them with dignified treatment that will ease their departure from this life, and offer them a way of spending their last days in peace

## Loneliness of the dying

The Western cultures promote fearing the process of death and dying. As the patient comes closer to death, the ultimate aloneness is endured; neither mortal nor faith in God can save the patient from death. Loneliness has been documented to be an integral part of ill health, for both the patient and even for the caregivers. The end-of-life experience encompasses feelings of hopelessness, death anxiety, fear, guilt, doubt, and loneliness. El-Jawahri et al.^4^ found that dying people who were aware of their terminal diagnosis rated their quality of their life as lower and showed a significant increase of emotional distress and anxiety. Consequently, they experienced feelings of emotional isolation and loneliness, starting to feel a loss of contact with the world, and these feelings are exacerbated when the person is institutionalized.^5^ What contributes to those feelings is that the dying person feels that no one can truly understand the situation or understand what it is to die. This deepens the feeling of aloneness and loneliness in the face of death. Additionally, it was observed that as one becomes terminally ill, one begins to lose their identity and sense of self. Whatever made them unique throughout their life, begins to fade. Third, as overwhelming loneliness engulfs the dying individual, there is a growing awareness that, as one becomes disconnected from one’s social world and others who may feel shut out of their life, one is left to die alone.^6^ As one’s physical limitations increase, and treatment takes its toll, the emotional distress that those terminally ill patients struggle with, contribute to the progressive isolation from others which leads to profound feelings of loneliness.^7^ While most of us wish that we could die at home, surrounded by our loved ones, most terminally ill people die in hospital beds, a manner of dying which ushers in loneliness, isolation, and a feeling that no one can truly understand them.

## To conclude

Loneliness, just like pain, joy, and sorrow, is an integral part of life, pronounced at times of illness, psychological distress, and of course death. We cannot escape loneliness, but we may wish to minimize its occurrence, intensity, and frequency. Only lately has loneliness started to “come out of the closet,” with COVID having contributed to minimizing the stigma that was attached to the lonely, so that the media was encouraged to address that issue, in addition to researchers. I believe that open discussion of loneliness, starting in grade one, is imperative to creating a society which can address it in the best and most efficient manner.

Research could assist in understanding and addressing loneliness, especially for those who need palliative care. For instance, carers (both family members, and paid ones) need to be informed of the occurrence of loneliness at the end of life, and understand its causes. Further, it would be advantageous for research to discover what can ease the loneliness of the dying, and whether preparations for death and the accompanying loneliness need to begin during our lives, rather than when it is close to its termination. And it may particularly enrich our understanding of loneliness in death, if research efforts can be directed at assisting the dying by finding a quick and easy way of training family members and carers in alleviating the isolation that the dying often feel.

## References

[1] Ernst M Niederer D Werner AM , et al. Loneliness before and during the COVID-19 pandemic: a systematic review with meta-analysis. Am Psychol 2022; 77(5): 660–677. 35533109 10.1037/amp0001005 PMC9768682

[2] Rokach A . The psychological journey to and from loneliness: development, causes, and effects of social and emotional isolation. Cambridge, MA: Academic Press, 2019.

[3] Abel J Kellehear A . Public health palliative care: reframing death, dying, loss and caregiving. Palliat Med 2022; 36(5): 768–769. 35531662 10.1177/02692163221096606

[4] El-Jawahri A Traeger L Park ER , et al. Associations among prognostic understanding, quality of life, and mood in patients with advanced cancer. Cancer 2014; 120(2): 278–285. 24122784 10.1002/cncr.28369

[5] Kuhl D . Exploring the lived experience of having a terminal illness. J Palliat Care 2011; 27(1): 43–52. 21510131

[6] Kellehear A . The inner life of the dying person. New York, NY: Columbia University Press, 2014.

[7] Rokach A Findler L Chin J , et al. Cancer patients, their caregivers and coping with loneliness. Psychol Health Med 2013; 18(2): 135–144. 22640693 10.1080/13548506.2012.689839

